# Potentially inappropriate prescribing in middle-aged adults: a significant problem with a lack of action and evidence to address it

**DOI:** 10.3399/BJGPO.2023.0209

**Published:** 2025-01-15

**Authors:** Michael Naughton, Frank Moriarty, Patrick Redmond

**Affiliations:** 1 The Clinical Effectiveness Group, Wolfson Institute of Population Health, Queen Mary University of London, London, UK; 2 School of Pharmacy and Biomolecular Sciences, RCSI University of Medicine & Health Sciences, Dublin, Republic of Ireland; 3 Department of General Practice, RCSI University of Medicine & Health Sciences, Dublin, Republic of Ireland

**Keywords:** Potentially inappropriate prescribing, middle-aged adults, medicines optimisation, primary healthcare, general practitioners

Potentially inappropriate prescribing (PIP), prescribing where the potential harms outweigh the potential benefits, or where a medication that a patient would benefit from is not prescribed, is an important healthcare challenge. PIP has been well characterised among older adults and is linked to adverse drug reactions (ADRs), hospitalisations, and increased healthcare costs.^1^ While studies have been conducted to address PIP in older adults, middle-aged adults remain overlooked despite also being vulnerable to PIP due to age-related chronic conditions.^2^

Our recently published systematic review showed that PIP is common in middle-aged adults, with an estimated 38% being exposed to PIP annually.^3^ PIP in middle-aged adults is known to occur in higher risk and disadvantaged groups: those with multimorbidity, polypharmacy, and those from deprived areas.^4^ It has been shown to be associated with ADRs,^5^ and may be associated with increased healthcare utilisation.^6^ A further study by our team, examined the cost of PIP in 1.2 million middle-aged adults in South London, finding that the total cost of PIP in this age group across 6 years was £2.8 million. The cost of adequate alternative prescribing would be £2.2 million, a cost-saving of approximately £553 874 compared with PIP.^7^

Following on from these studies, we conducted a further systematic search (unpublished) to examine interventions to reduce this prescribing. Searches were conducted in MEDLINE, EMBASE, CINAHL, Cochrane library, ProQuest, Web of Science, OpenGrey, Clinicaltrials.gov, and the WHO Clinical Trials Registry Platform. All English language studies that included adults aged 45–64 years, applied explicit PIP criteria, implemented an intervention to reduce PIP, and were published by June 2022, were eligible. In total, 12 384 studies underwent title and abstract screening, with 248 articles identified for full text screening, however ultimately none met our inclusion criteria.

Our search has revealed a literature gap, with no studies having been conducted with interventions aiming to reduce PIP in middle-aged adults. Conversely, there are numerous interventional studies to reduce PIP in older adults.^8,9^ PIP in older adults has a similar prevalence,^10^ but in absolute terms the largest burden of PIP exists in middle-aged adults, due to the larger population size. Intervening earlier in middle age may allow patients’ medicines to be optimised and avoid adverse outcomes as they age.

Furthermore, the benefits of targeting high risk prescribing independent of age, rather than concentrating only on older adults, have been demonstrated by multiple studies. Concentrating on high risk prescribing across all age groups, these studies have shown interventions can reduce high risk prescribing, and associated adverse outcomes such as gastrointestinal bleeds, heart failure, and hospital admissions.^11^ The PINCER intervention has also shown that interventions to reduce high risk prescribing can be highly cost effective.^12^ The current, extremely welcome, deprescribing initiatives (https://deprescribing.org/) are applicable beyond older adults and could also be used to benefit the middle-aged in particular. Therefore, as well as extending interventions to middle-aged people specifically, it is also worth considering a whole population approach to high risk prescribing or PIP, given the demonstrated successes and cost effectiveness of these approaches previously.

As practising clinical academics, we are concerned about the lack of policy and research activity to develop interventions to reduce PIP in middle-aged adults. This is an issue affecting a significant proportion of the middle-aged population and it is vital to understand how to reduce this prescribing to avoid preventable harms and unnecessary cost to the health service. I urge primary care journals, including BJGP Open, to prioritise the issue of appropriate prescribing outside of the narrow focus on older adults by encouraging submissions and facilitating discourse among researchers, practitioners, and policymakers. This would contribute to our understanding of PIP in other age groups, including middle-aged adults, and help to develop interventions to address the issue in wider patient groups. I hope this article serves as a catalyst for discussion and research on this pressing issue.

## Data

N/A
